# Conducting composite materials from the biopolymer kappa-carrageenan and carbon nanotubes

**DOI:** 10.3762/bjnano.3.48

**Published:** 2012-05-23

**Authors:** Ali Aldalbahi, Jin Chu, Peter Feng, Marc in het Panhuis

**Affiliations:** 1 Soft Materials Group, School of Chemistry, and ARC Centre of Excellence for Electromaterials Science, University of Wollongong, Wollongong, NSW 2522, Australia; 2 Institute of Functional Nanomaterials and Department of Physics, University of Puerto Rico, San Juan, Puerto Rico 00931, USA

**Keywords:** biopolymers, carbon nanotubes, carrageenan, composite materials, conductivity, mechanical, rheology

## Abstract

Conducting composite films containing carbon nanotubes (CNTs) were prepared by using the biopolymer kappa-carrageenan (KC) as a dispersant. Rheological studies indicated that 0.5% w/v was the appropriate KC concentration for dispersing CNTs. Our results showed that multiwalled nanotubes (MWNTs) required less sonic energy than single-walled nanotubes (SWNTs) for the dispersion process to be complete. Films prepared by vacuum filtration exhibited higher conductivity and improved mechanical characteristics compared to those prepared by evaporative casting. All composite films displayed sensitivity to water vapour, but MWNT films were more sensitive than SWNT films.

## Introduction

Carbon nanotubes (CNTs) have attracted attention due to their unique electronic, mechanical, optical and thermal properties, which make them suitable for applications in nanotechnology [1–4]. However, one of the main disadvantages of CNTs is their process-ability; they are difficult to disperse in most common solvents due to their high surface energy and van der Waals interactions [3,5–7]. To overcome this issue, a diverse range of molecules have been used to aid the dispersion of CNTs in aqueous media, such as surfactants, polymers and biopolymers [8–16]. Well known examples of surfactants and polymers include, sodium dodecyl sulfonate, Triton X-100 and polystyrene sulfonate [17–24]. In addition, it has been established that biopolymers such as gellan gum, xanthan gum, gum arabic and iota-carrageenan are effective for the dispersion of CNTs in aqueous solutions [8,25–29]. For example, gellan gum-CNT dispersions have been wet-processed by inkjet printing into optically transparent films, which displayed sensitivity to water vapour [30].

Other commonly employed wet-processing methods used to process biopolymer–CNT dispersions into materials include (but are not limited to) evaporative casting, vacuum filtration and fibre spinning [11,29]. Formation of films by evaporation is well-known and involves the controlled evaporation of the solvent from a CNT dispersion. It has been established that the mechanical and electrical characteristics of these CNT networks are contingent on the CNT/dispersant ratio. Increasing the nanotube concentration usually leads to an increase in the electrical conductivity and to mechanical reinforcement [31–32]. Vacuum filtration of dispersions usually results in films, which are generally referred to as buckypapers [9,33]. These films can be defined as an entangled network of CNTs, which are held together by van der Waals interactions at the CNT–CNT junctions and are arranged in a two-dimensional structure [34]. Although the formation of buckypapers is straightforward, it has been shown that the electrical, mechanical and physical characteristics are dependent on various parameters, such as the type of CNTs (SWNT or MWNT), the filtration substrate (pore size; hydrophilic or hydrophobic), the sonication time and the type of dispersant (surfactants or polymers) [9,33]. The electrical properties combined with their flexible nature makes CNT networks ideal for a number of potential applications, such as solar cells, displays, touch screens, sensors, electronic paper, supercapacitors and batteries [35–38].

Carrageenans are a biopolymer family of water-soluble, linear, sulfonated galactans extracted from various sources of the Rhodophyta (marine red algae). The carrageenans are well known for their gel-forming and thickening properties [39–40]. This biopolymer is an anionic polysaccharide whose structure contains galactose, 3,6-anhydrogalactose units, carboxy and hydroxy groups and ester sulfates. There are three types of carrageenan depending on the number of charged sulfated groups per biopolymer repeat unit, i.e., kappa-carrageenan (one group), iota-carrageenan (two groups) and lambda-carrageenan (three groups) [39]. Carrageenans have been extensively employed in the food industry and are commonly referred to as E407 (European Union specification) as well as being approved by the US Food and Drug Administration as a direct food additive [40]. Recent demonstrations of other applications include their use in drug delivery for the inhibition of viral infections [41–42].

Glycerin (or glycerol, glycerine) is a polyol compound widely used in a diverse range of industries. For example, in the food industries it is added as a humectant, while it is also used to produce an essential ingredient (glyceryl nitrate) for explosives. Of particular relevance to the research presented in this paper is its usage as a plasticizer to increase the flexibility of polymer films [43].

In this work, it is shown that kappa-carrageenan (KC) is a suitable dispersant for the stabilization of SWNTs and MWNTs in water. The KC concentration and sonication time were optimised to facilitate the efficient dispersion of these CNTs. The electrical and mechanical characteristics of free-standing composite films prepared by evaporative casting and vacuum filtration were assessed, including the effect of incorporating the plasticizer glycerin. The gas-sensing ability of these composite films is demonstrated.

## Results and Discussion

### Rheological of carrageenan solutions

**Figure 1:**
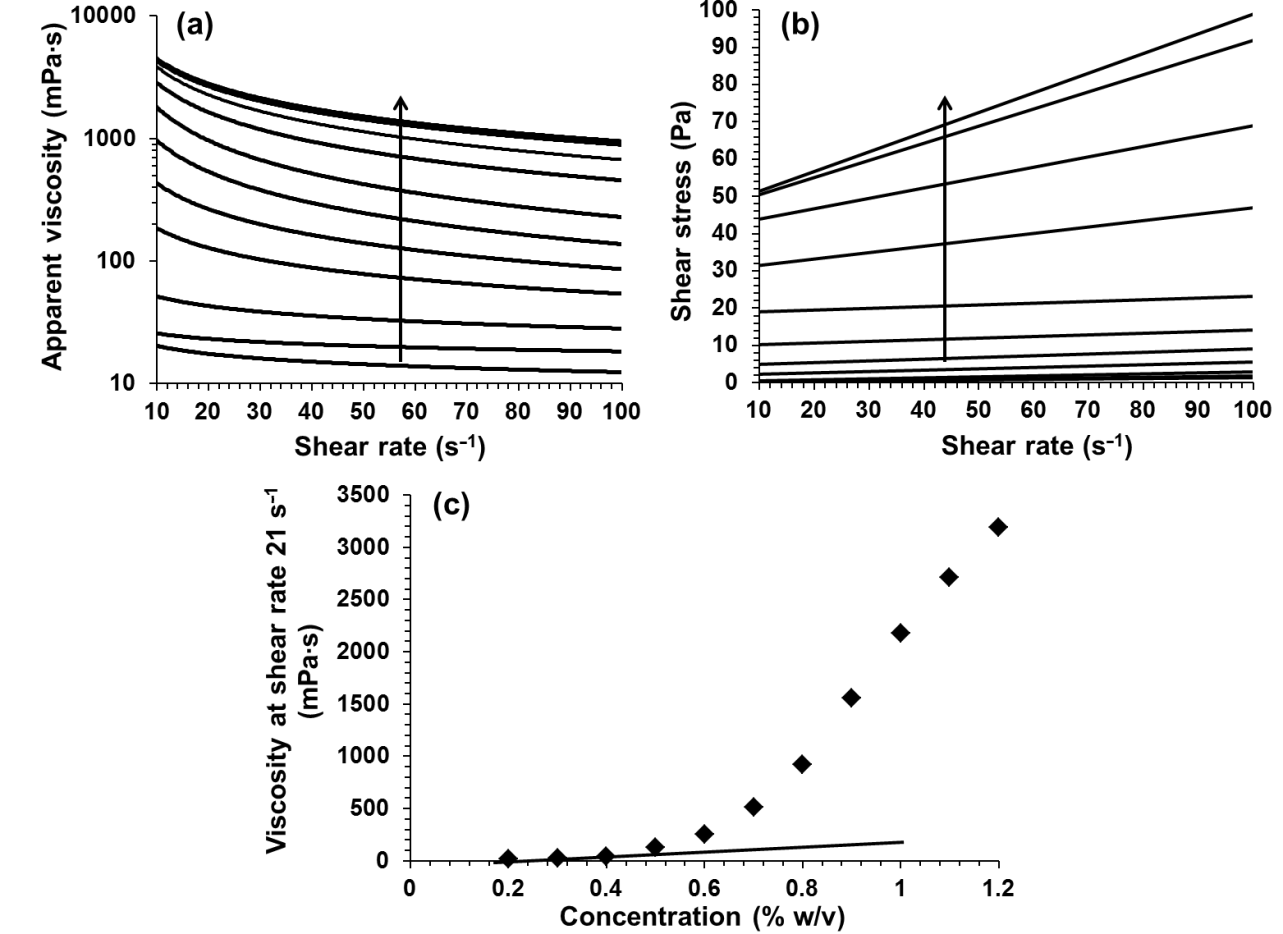
Effect of increasing concentration on (a) the viscosity and (b) the shear stress versus shear rate of KC solutions. The lines in (a) and (b) are fits to Equation 1 and Equation 2, respectively (measured data points omitted for clarity). The arrows indicate the direction of increase in KC concentration (0.2–1.2% w/v). (c) Viscosity at shear rate 21 s^−1^ as a function of concentration of KC. All samples were measured at 21 °C. The straight line in (c) indicates the rate of increase at the lower concentrations.

Rheology is a suitable method for following any changes in viscosity of gel-forming polymers, such as the carrageenans. This is an important step due to the adverse effect that the viscosity of a solution can have on the sonication process. Polymers undergo a dilute to semidilute transition resulting in a significant change in their viscosity. High viscosity is undesirable as it decreases CNT mobility, which impedes the efficiency of the dispersion process. Therefore, our initial studies focussed on establishing the appropriate biopolymer concentration using flow-curve analysis. The viscosity was measured as a function of shear rate for KC solutions over a concentration range of 0.2–1.2% w/v at 21 °C (Figure 1a). All KC solutions displayed shear-thinning behaviour, i.e., decreasing viscosity (η) with increasing shear rate (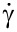). These flow curves were fitted to the well-known power-law model [44]:

[1]
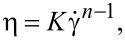


where *K* and *n* indicate the “consistency” and power-law index, respectively. Figure 1a shows that the viscosity of KC solutions increased with increasing concentration. For example, the apparent viscosity of the KC solution (at shear rate 21 s^−1^) increased from 16 mPa·s at 0.2% w/v to 3190 mPa·s at 1.2% w/v; whereas, the consistency of KC exhibited an increase from 33 ± 1 mPa·s*^n^* to 21890 ± 48 mPa·s*^n^* as the concentration was increased from 0.2 to 1.2% w/v. This behaviour is consistent with observations of other polysaccharides [45–46] and polymers in general [47].

Table 1 shows that KC solutions with a concentration <0.5% w/v have power-index values of ~0.8. However, for higher concentrations the solutions become more shear-thinning (*n* decreases), and thicker (*K* increases). Figure 1c shows a sharp increase in the apparent viscosity of the KC solution around 0.5% w/v, which is characteristic of dilute to semidilute transition.

**Table 1:** Summary of rheology analysis of KC solutions at different concentrations (*c*). Consistency (*K*) and power-law index (*n*) values were obtained through curve fitting with the power-law model (Equation 1). Bingham yield point (τ_B_) and Bingham flow coefficient (η_B_) values were obtained by using the Bingham model (Equation 2). Values for all solutions were obtained over a shear-rate range of 10–100 s^−1^ and 21 °C.

*c* (% w/v)	*K* (mPa·s*^n^*)	*n*	τ_B_ (Pa)	η_B_ (Pa·s)

0.2	33 ± 1	0.79 ± 0.01	0.11 ± 0.01	0.012 ± 0.001
0.3	36 ± 1	0.85 ± 0.01	0.11 ± 0.01	0.018 ± 0.001
0.4	94 ± 1	0.74 ± 0.01	0.37 ± 0.01	0.025 ± 0.001
0.5	637 ± 1	0.46 ± 0.01	1.88 ± 0.05	0.037 ± 0.001
0.6	2158 ± 2	0.30 ± 0.01	4.49 ± 0.06	0.044 ± 0.001
0.7	6725 ± 9	0.16 ± 0.01	9.87 ± 0.05	0.044 ± 0.001
0.8	14030 ± 12	0.11 ± 0.01	18.53 ± 0.18	0.047 ± 0.001
0.9	17718 ± 6	0.21 ± 0.01	29.79 ± 0.39	0.173 ± 0.006
1.0	20506 ± 42	0.24 ± 0.01	41.08 ± 0.89	0.279 ± 0.015
1.1	21058 ± 32	0.32 ± 0.01	45.78 ± 1.21	0.462 ± 0.019
1.2	21890 ± 48	0.33 ± 0.01	46.08 ± 0.54	0.529 ± 0.008

The relation between shear stress and shear rate for IC solutions at different concentrations is shown in Figure 1b. It can be seen that KC solutions exhibit a yield point, i.e., the viscous KC solutions start to flow only when a certain amount of force is applied. This point can be determined by using approximations such as the Bingham model [44]:

[2]
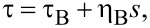


where τ_B_ and η_B_ indicate the Bingham yield point and Bingham flow coefficient, respectively. Although the values obtained by using the Bingham model are dependent on the shear-rate range, it provides a good approximation for the determination of yield points [44]. The model shows that, over a shear-rate range of 10–100 s^−1^, the Bingham yield point and Bingham flow coefficient significantly increased with concentration. For example, the Bingham yield point of the KC solution (0.2% w/v) was 0.11 ± 0.01 Pa compared to 46.08 ± 0.54 Pa at a higher concentration (1.2% w/v), as shown in Table 1. Thus, it is clear that an increase in concentration results in an increase in Bingham yield point and Bingham flow coefficient.

**Figure 2:**
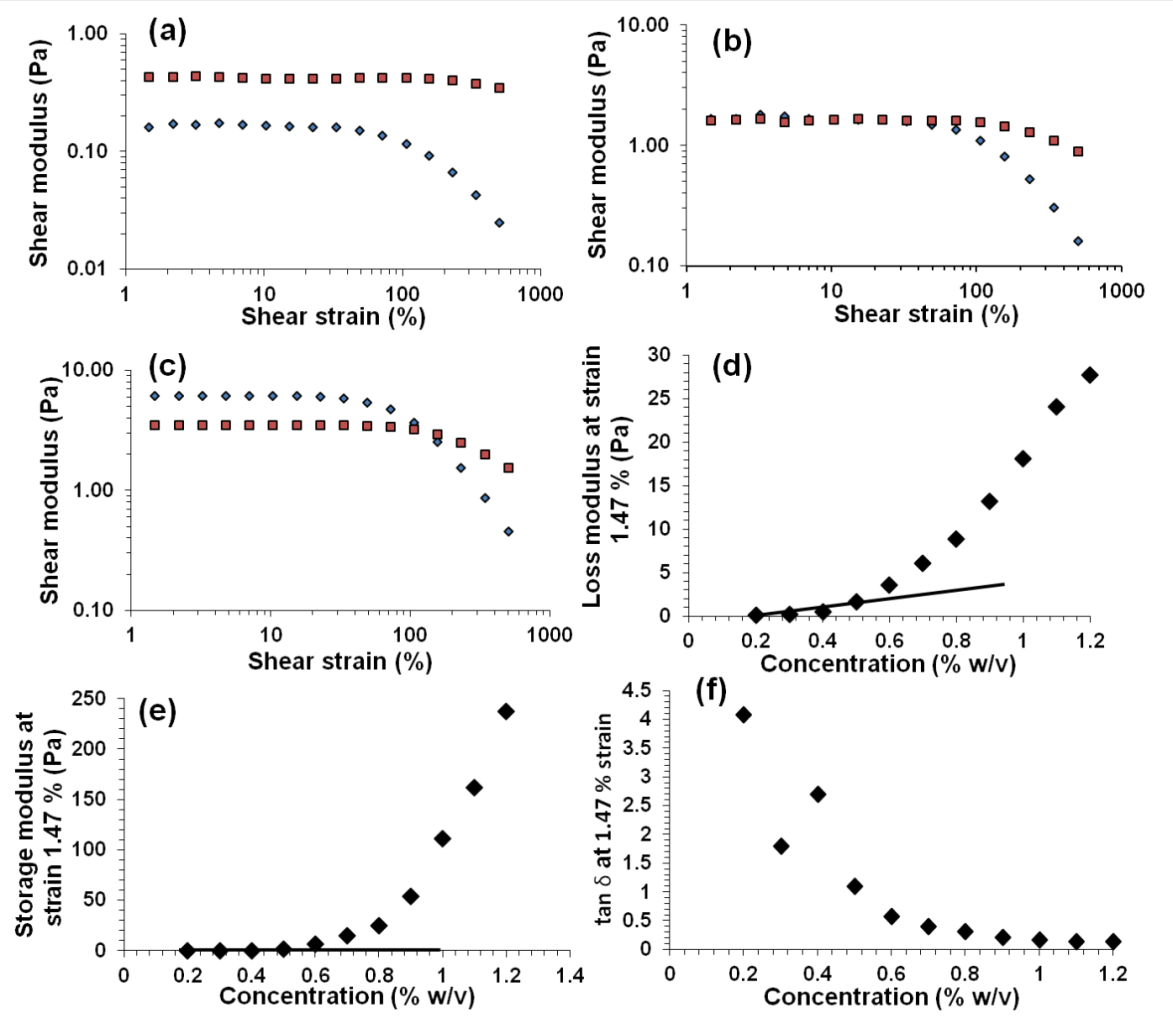
(a–c) Storage (G΄, diamonds) and loss modulus (G˝, squares) of KC solutions at concentrations of 0.4%, 0.5%, and 0.6% w/v, respectively; (d and e) loss and storage modulus of KC versus solution concentration at 1.47% shear strain, and (f) loss factor (tan δ = G˝/G΄) versus concentration for KC at 1.47% shear strain. The straight lines in (d and e) indicate the rate of increase at the lower concentrations.

It is well-known that rheology through dynamic modulus measurements can be used to determine the sol–gel transition of polymer solutions. A larger loss modulus (G˝) than storage modulus (G΄) in the linear viscoelastic region is indicative of solution-like behaviour. Whereas, the reverse (G΄ > G˝) is indicative of gel-like behaviour [44]. The KC solutions with concentrations below 0.5% w/v exhibited lower G΄ values than G˝ values (Figure 2a–c). As expected, by increasing the concentration, the loss and storage moduli increased (Figure 2d–e), but two distinct rates of increase were observed. Figure 2f shows this data expressed in terms of the loss factor, tan δ = G˝/G΄ at a fixed shear-strain value (1.47%). Values of tan δ > 1 indicate solution-like behaviour, whereas tan δ values ≤ 1 point towards gel-like behaviour. These results provide further evidence for a dilute to semidilute transition for KC concentrations around 0.5% w/v.

### Optimisation of sonication time

A KC concentration (0.5% w/v) in the dilute range was selected to optimise the dispersion of CNTs at a concentration of 0.10% w/v. The optimum sonication time is defined as the minimum amount of time required to successfully disperse the CNTs [8]. This optimisation is necessary as it has been reported that excess sonication leads to damage of the nanotubes [15,48]. The optimum sonication time was determined as defined in [8], by establishing the time it takes for the UV–vis absorption intensity to level out and the visible aggregates to disappear. CNTs absorb at most wavelengths, while KCs do not exhibit any bands for wavelengths greater than 250 nm; thus, by monitoring a wavelength in this range the dispersion of CNTs can be monitored.

**Figure 3:**
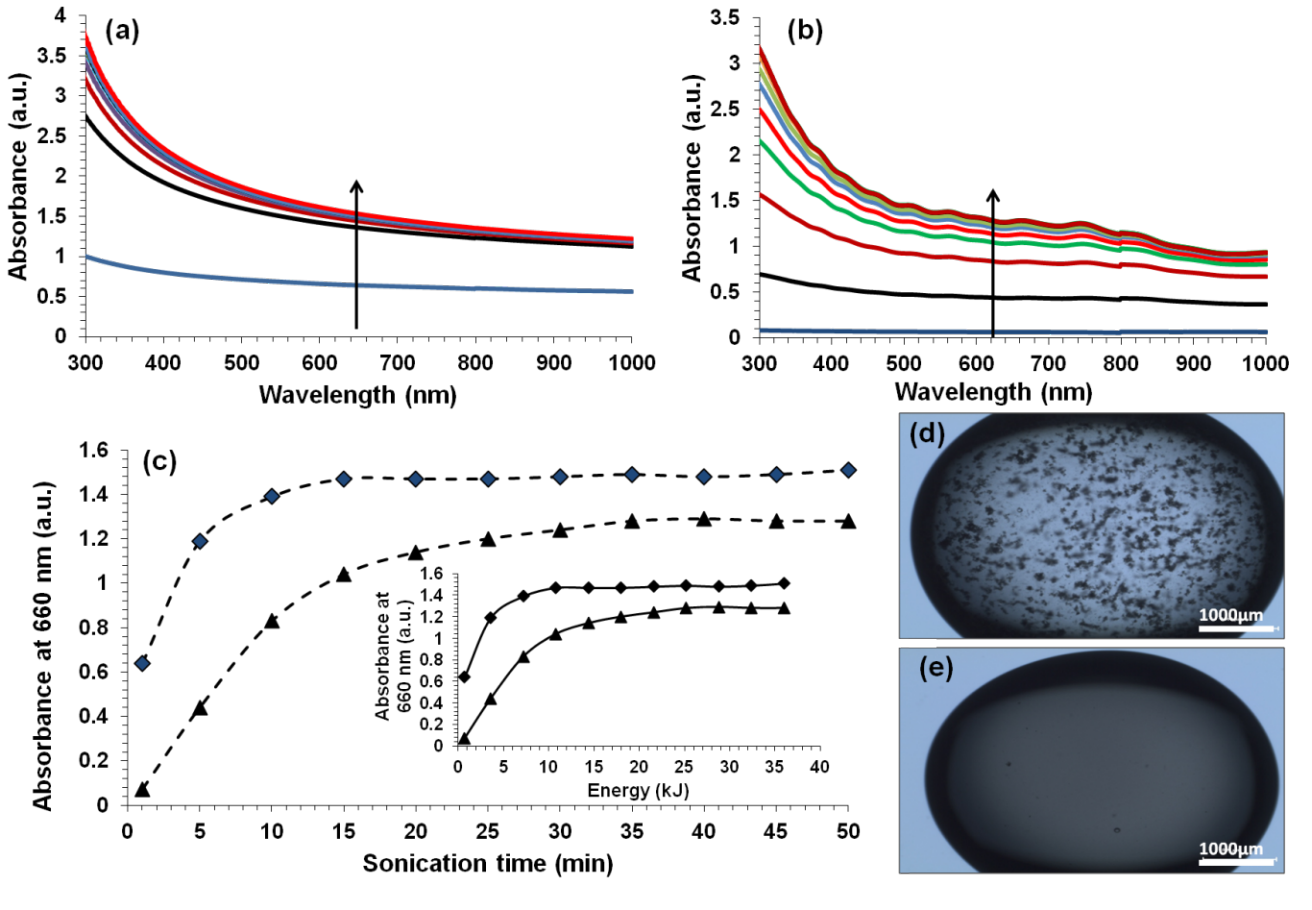
Effect of increasing sonication time on the UV–visible absorption spectrum of a dispersion containing (a) 0.10% w/v MWNTs and 0.50% w/v KC and (b) 0.10% w/v SWNTs and 0.50% w/v KC. (c) Absorbance at 660 nm versus sonication time and energy (inset) for KC–MWNT (diamonds) and KC–SWNT (triangles) dispersions. (d and e) KC–SWNTs after 5 and 35 minutes sonication, respectively. All samples were measured at 21 °C. Arrows indicate increasing sonication time.

Figure 3a and Figure 3b show that the UV–vis absorbance intensity increases with sonication time, indicating that an increasing amount of CNTs became dispersed over time. The absorbance at an arbitrarily picked wavelength (660 nm) becomes independent of sonication after 20 and 35 min of sonication for MWNTs and SWNTs, respectively (Figure 3c). Optical microscopy revealed the presence of aggregates in the dispersions subjected to short sonication times (5 min), see Figure 3d. In contrast, after 20 and 35 min of sonolysis no aggregates were visible, suggesting that homogenous dispersions were achieved. Therefore, these sonication times (20 and 35 minutes) were selected as being optimal for ensuring that the MWNTs and SWNTs were well dispersed in the KC solution. Conversion of sonication time to energy shows that achieving complete dispersion of MWNTs and SWNTs requires 14.4 ± 0.8 kJ (~0.96 kJ per mg) and 25.2 ± 1.1 kJ (~1.68 kJ per mg), respectively (inset in Figure 3c), i.e. SWNTs are 1.75 times more costly to disperse than are MWNTs.

### Stability and rheology of optimised dispersions

**Figure 4:**
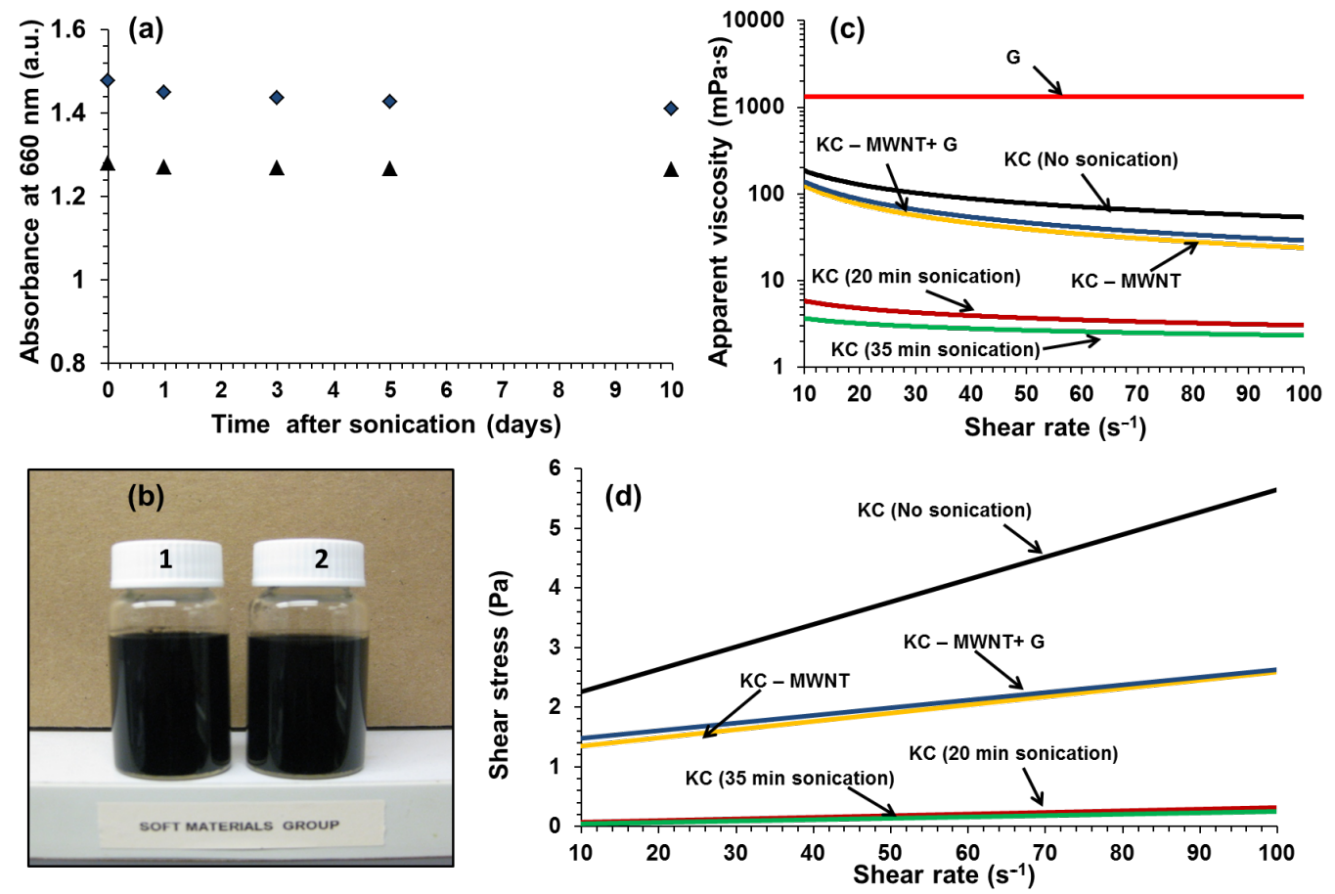
(a) UV–vis absorbance at 21 °C and at 660 nm wavelength for MWNT (diamonds) and SWNT (triangles) dispersions as a function of time. (b) Photographs of KC–MWNT (1) and KC–SWNT (2) dispersions after being left undisturbed for two months. (c) Apparent viscosity as a function of shear rate for undiluted glycerin (G), KC solutions (0.5% w/v) at different sonication times and KC–CNTs (KC concentration = 0.5% w/v, CNT concentration = 0.1% w/v) dispersions. (d) Shear stress versus shear rate of unsonicated KC, at different sonication times of KC solutions and KC–CNTs dispersions. The lines in (c) and (d) are fits to Equation 1 and Equation 2, respectively.

Wet-processing methods, such as vacuum filtration and evaporative casting, require dispersions that are stable for several days. Stability was assessed by monitoring of the UV–vis absorbance as a function of time. Figure 4a shows that the KC–CNT dispersions are reasonably stable for a period of at least 10 days. In addition, these dispersions appeared to be stable after two months of storage under controlled conditions (21 °C, RH = 45%, Figure 4b).

The flow curves of KC–CNT dispersions and sonicated KC solutions are shown in Figure 4b and Figure 4c. It is clear that the apparent viscosity and consistency of KC solutions decreased significantly during sonication, while the value of the power-law index increased (Table 2). For example, over 35 minutes of sonication the consistency decreased from 637.4 ± 4.4 mPa·s*^n^* to 5.8 ± 0.1 mPa·s*^n^*, while *n* increased from 0.46 to 0.80. This suggests that sonication results in solutions that are thinner (*K* decreases) and less shear-thinning (*n* increases). This is in excellent agreement with previous observations, i.e., sonolysis reduces the molecular weight of the biopolymer, and this is responsible for the observed reduction in apparent viscosity [8,11,49]. The addition of CNTs resulted in dispersions that were thicker (*K* increases) and more shear-thinning (*n* decreases) than the corresponding sonicated KC solutions (Table 2). Similar observations were made for the Bingham parameters, i.e., sonolysis reduced the τ_B_ and η_B_ values, while the addition of CNT resulted in increased values. As expected, the addition of glycerin did not dramatically affect the flow properties of the KC-CNT dispersions. Glycerin is a Newtonian fluid, i.e., *n* ~ 1 indicating that its viscosity is independent of the shear rate (Table 2).

**Table 2:** Summary of rheological analysis over a shear-rate range of 10–100 s^−1^ at 21 °C for KC solutions, and KC–CNT and KC–CNT–G dispersions for different sonication times (ST). Concentrations of KC, CNT and G are 0.5% w/v, 0.10% w/v and 0.25% w/v, respectively. Consistency (*K*) and power-law index (*n*) values were obtained through curve fitting with the power-law model (Equation 1). Bingham yield point (τ_B_) and Bingham flow coefficient (η_B_) values were obtained using the Bingham model (Equation 2).

Sample	ST (min)	*K* (mPa·s*^n^*)	*n*	τ_B_ (Pa)	η_B_ (Pa·s)

KC	0	637 ± 4	0.46 ± 0.01	1.88 ± 0.17	0.047 ± 0.003
KC	20	11.2 ± 0.2	0.72 ± 0.01	0.04 ± 0.01	0.003 ± 0.001
KC	35	5.8 ± 0.1	0.80 ± 0.01	0.02 ± 0.01	0.002 ± 0.001
G	0	1320.0 ± 0.1	0.99 ± 0.01	0.19 ± 0.02	1.314 ± 0.002
KC–MWNT	20	648.5 ± 4.4	0.28 ± 0.01	1.21 ± 0.02	0.014 ± 0.001
KC–MWNT–G	20	662.9 ± 5.7	0.32 ± 0.01	1.35 ± 0.01	0.013 ± 0.001
KC–SWNT	35	814.4 ± 4.4	0.21 ± 0.01	1.29 ± 0.01	0.010 ± 0.001
KC–SWNT–G	35	849.4 ± 8.5	0.26 ± 0.01	1.26 ± 0.01	0.014 ± 0.001

### Electrical conductivity of films

**Figure 5:**
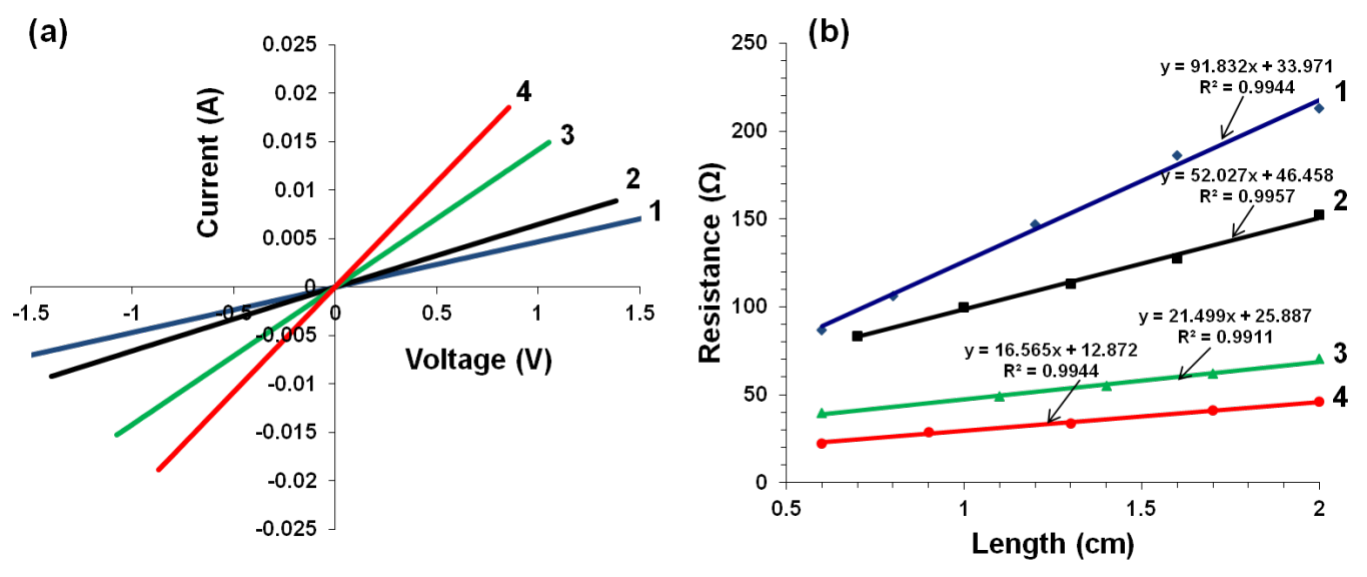
(a) *I*–*V* characteristics for KC–CNT (channel length 2 cm) and (b) resistance as a function of length for KC–CNT composite films prepared by evaporative casting and vacuum filtration of KC–CNT dispersions. Numbers 1 and 2 are KC–MWNT and KC–SWNT composite films, respectively, prepared by evaporative cast method. Numbers 3 and 4 are KC–MWNT and KC–SWNT composite films, respectively, prepared by the vacuum-filtration method. The straight lines in (b) are fits to Equation 3.

Free-standing films were prepared by evaporative casting and vacuum filtration of KC–CNT dispersions. All films exhibited linear *I–V* characteristics, i.e., ohmic behaviour (Figure 5a). The total resistance (*R*_T_) increased with channel length (Figure 5b), and was found to scale linearly with sample length according to [9,27]

[3]
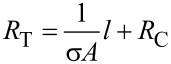


where *l*, *A*, σ and *R*_C_ are the length, cross-sectional area, electrical conductivity and contact resistance of the sample, respectively. The slope of the straight-line fit to Equation 3 can then be used to calculate the bulk conductivities (Table 3). Due to the difference in the density values of MWNTs (2.15 g/cm^3^) and SWNTs (1.5 g/cm^3^) it is not appropriate to compare in terms of mass fraction, but rather the volume fraction is more suitable. The CNT mass (*M*_f_) and volume (*V*_f_) fractions of films prepared by evaporative casting were obtained as follows:

[4]
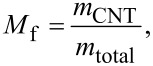


[5]
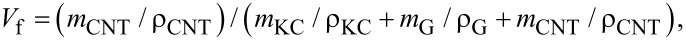


where *m*_CNT_, *m*_KC_, *m*_G_, *m*_total_*,* ρ_CNT_, ρ_KC_*,* and ρ_G_, are the mass of CNT, KC, and G, their total mass, and the densities of CNT, KC and G, respectively. The density value of KC was determined experimentally (1.22 ± 0.06 g/cm^3^) and the well-known density values of G (1.26 g/cm^3^) and the CNTs were used to calculate the CNT volume fraction. It was found that evaporation-cast MWNT films exhibited higher conductivity values compared to SWNT films at a similar volume fraction, *V*_f_ ~ 0.10. The conductivity of SWNT films with a higher volume fraction (*V*_f_ = 0.13) was still lower than that of a MWNT film with *V*_f_ = 0.10. These observations are in agreement with our previous observations for biopolymer composite materials [8].

**Table 3:** Effect of preparation method and addition of glycerin (G) on the conductivity (σ) of KC–CNT films prepared by evaporative casting (E1–4) and vacuum filtration (B1–4) methods. CNT mass (*M*_f_) and volume (*V*_f_) fractions values are calculated by using Equation 4 and Equation 5, respectively. The naming of the dispersions indicates the concentrations of biopolymer, CNTs and glycerin, i.e., “KC05–MW01–G025” corresponds to dispersion with KC, MWNT and G concentrations of 0.5% w/v, 0.1% w/v and 0.25% w/v, respectively.

Film	Dispersion	θ	*M*_f_	*V*_f_	σ (S/cm)

E1	KC05–MW01	64.5 ± 1.1	0.17	0.10	8.6 ± 1.6
E2	KC05–MW01–G025	56.0 ± 1.1	0.12	0.071	5.0 ± 0.9
E3	KC05–SW01	62.7 ± 1.1	0.17	0.13	7.4 ± 0.9
E4	KC05–SW01–G025	50.9 ± 1.4	0.12	0.099	2.9 ± 0.5
B1	KC015–MW003	76.9 ± 0.8	—	—	16.4 ± 1.6
B2	KC015–MW003–G0075	72.4 ± 0.8	—	—	14.5 ± 1.7
B3	KC015–SW003	79.5 ± 2.0	—	—	25.4 ± 1.6
B4	KC015–SW003–G0075	73.0 ± 0.8	—	—	17.9 ± 1.9

**Figure 6:**
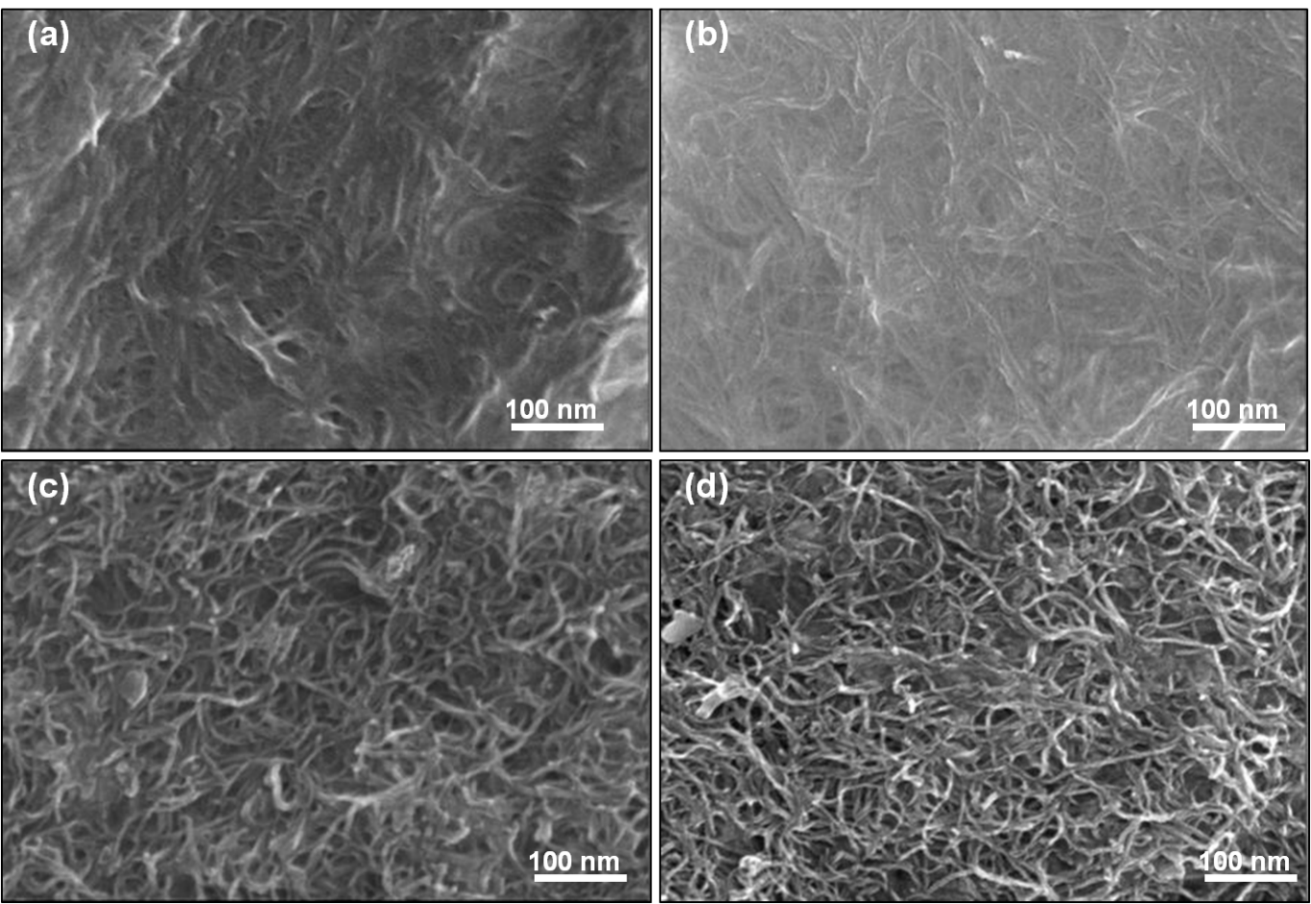
SEM image of (a) KC–CNT and (b) KC–CNT–G composite films prepared by the evaporative-casting method. (c) KC–CNT and (d) KC–CNT–G composite films prepared by the vacuum-filtration method. Contact angle values for (a–d) are 64°, 56°, 77° and 72°, respectively.

It was not possible to calculate the CNT mass or volume fractions for buckypapers, as it is unknown what was lost during the filtration process. In our previous work, we showed that the contact angle increases linearly with CNT mass and volume fraction [8]. The contact angle of all buckypaper materials is higher than those of evaporation-cast films (Table 3). This could suggest that the CNT mass/volume fraction in the buckypapers is higher than those of the evaporation-cast samples. This is supported by the difference in the surface morphology as observed in SEM images (Figure 6), i.e., the biopolymer coverage of the CNTs is more extensive for evaporation-cast films than for buckypapers. The lower degree of coverage can be attributed to the partial removal of KC and CNTs during the vacuum filtration process. These observations are supported by the difference in conductivity between casted (7.4 S/cm) and buckypaper (25.4 S/cm) SWNT composite films, with similar results for MWNT composite films. Hence, it is clear that the partial removal of KC results in an increase in the conductivity. The conductivity values of KC–SWNT buckypapers (25.4 ± 1.6 S/cm), were higher compared to those of the KC–MWNT buckypapers (16.4 ± 1.6 S/cm).

Incorporation of the hydrophilic plasticizer glycerin in the composite films reduced their conductivity and contact angle values. For example, the conductivity of a KC–SWNT film prepared by the evaporative-casting method decreased from 7.4 S/cm to 2.9 S/cm through the addition of glycerin. This lowering of the conductivity suggests that glycerin may affect the number of conducting pathways or junctions in the nanotube network.

### Mechanical properties of films

**Figure 7:**
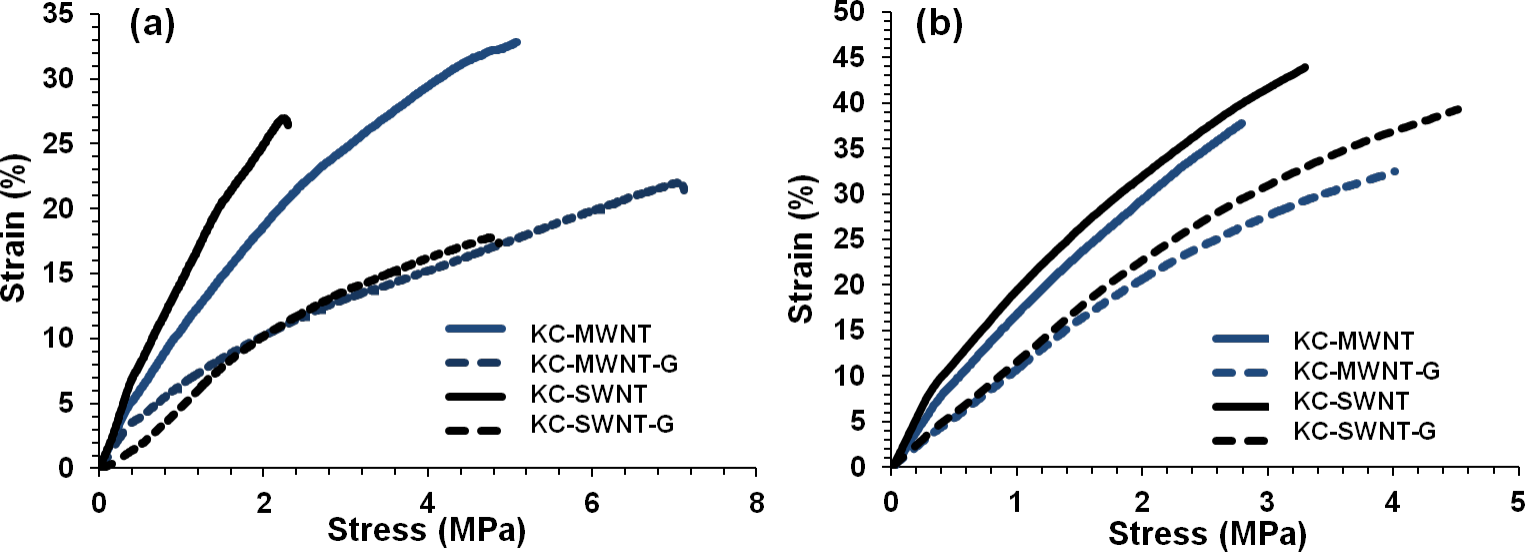
Stress–strain curves for films with and without glycerin prepared by (a) evaporative casting and (b) vacuum filtration methods.

The mechanical characteristics of the free-standing films prepared by evaporative casting and vacuum filtration of KC–CNT dispersions are shown in Figure 7. Sonication of the KC solution prior to film formation reduced the mechanical characteristics of these films. The sonication-induced reduction in the molecular weight resulted in films with reduced values of tensile strength (*TS* = 20 MPa), strain-at-break (γ = 2%) and Young’s modulus (*E* = 1165 MPa) (Table 4).

**Table 4:** Summary of the mechanical properties of composite films prepared by evaporative casting (E1–4) and vacuum filtration (B1–4). Young’s modulus (*E*), tensile strength (*TS*) and strain-at-break (γ). E1–4 and B1–4 refer to composite films listed in Table 3.

Film	*E* (MPa)	*TS* (MPa)	γ (%)

E1	1414 ± 43	32 ± 4	5.1 ± 0.7
E2	1031 ± 40	21 ± 2	7.1 ± 0.8
E3	1640 ± 45	27 ± 3	3.3 ± 0.5
E4	434 ± 29	18 ± 2	4.8 ± 0.6
B1	2184 ± 77	36 ± 3	2.5 ± 0.6
B2	1142 ± 61	32 ± 3	4.0 ± 1.0
B3	2848 ± 81	44 ± 4	2.3 ± 0.8
B4	1228 ± 49	39 ± 3	4.5 ± 1.0

The addition of CNTs resulted in increases in the *TS*, γ and *E* values for both MWNTs and SWNTs compared to the corresponding values for the sonicated KC film (Table 4). This can be attributed to the mechanical reinforcement effect of incorporating CNTs into the polymer matrix [32,50]. Films produced by the evaporative-casting method exhibited higher *E* and *TS* values compared to films produced by vacuum filtration. In contrast, films prepared by the evaporative-casting method exhibit higher strain-at-break values than do films produced by vacuum filtration. Hence, it is clear that films produced by vacuum filtration are more robust and less ductile compared to films prepared by the evaporative-casting method.

Incorporation of a plasticizer (glycerin) resulted in a reduction of the *E* and *TS* values but improved ductility. For example, the γ value for KC–MWNTs films with glycerin prepared by the evaporative-cast method is 7.1% compared to 5.1% for the same film without glycerin. This suggests that glycerin is a good material for improving the mechanical handleability of these CNT composite films.

### Sensing properties of films

**Figure 8:**
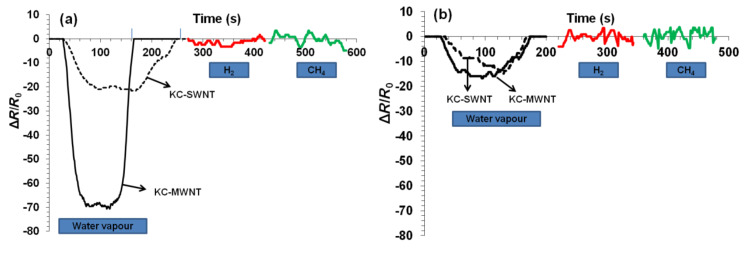
Response of KC–MWNT and KC–SWNT composite films to humidity change, H_2_ and CH_4_ gases (100 ppm in air) at operating temperature of 25 °C. Films prepared by (a) evaporative casting and (b) vacuum filtration.

The sensitivity (*S*) of films against humidified air, and H_2_ and CH_4_ gases was investigated by monitoring the resistance as a function of time [51]:

[6]
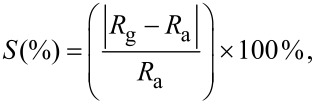


where *R*_a_ and *R*_g_ represent the resistance of the film before and during exposure to the target gas (humidified air, H_2_ and CH_4_), respectively. The sensitivity of the films to humidity was investigated over a relative humidity change from 40% to 90%. All films responded to the change in humidity, but it was not possible to detect any response after exposure to H_2_ and CH_4_ gases at 25 °C (Figure 8). The KC–MWNT films displayed higher sensitivity to water vapour compared to the corresponding KC–SWNT films. For example, the sensitivity of MWNT films was *S* = 70 ± 10% compared to *S* = 25 ± 5% for SWNT films. However, the response/recovery times were faster for MWNT films (50 s) compared to SWNT films (70 s). The sensitivity was significantly reduced upon incorporation of glycerin, e.g., from *S* = 70 ± 10% to *S* = 20 ± 5% for MWNT composite films. Buckypaper films displayed lower sensitivity values of ~17% (MWNT) and ~15% (SWNT), respectively. It is likely that the observed differences in sensitivity can be attributed to the processing methods, i.e., the vacuum filtration process results in partial removal of KC, as discussed above. It is not clear at present why MWNT films prepared by evaporative casting are about three times more sensitive compared to SWNT films. Further research is necessary to fully understand this.

## Conclusion

In this work, rheological analysis was used to determine the appropriate concentration (0.5% w/v) for dispersing SWNTs and MWNTs by using the biopolymer KC. It was shown that MWNTs required less sonication compared to SWNTs, i.e., a lower amount of energy input. Rheological analysis revealed that an increasing amount of sonolysis reduced the flow characteristics (viscosity) of KC solutions, while addition of CNTs increased viscosity.

KC–MWNT films prepared by an evaporative-casting process displayed higher conductivity compared to KC–SWNT films. As expected, the conductivity of all buckypaper films was higher than films prepared by evaporative casting. It was observed that the incorporation of CNTs in the polymer matrix resulted in an increase in the values of the mechanical properties. The addition of a plasticizer (glycerin) improved the mechanical handleability, but at the cost of electrical conductivity. Buckypaper films displayed superior electrical and mechanical characteristics (bar ductility) over evaporation-cast films, but they were less sensitive to changes in the humidity. MWNT films exhibited sensitivity to humidity as high as of 70%, easily outperforming SWNT films. This work contributes toward the development of conducting biopolymer composite materials.

## Experimental

### Materials

The biopolymer iota-carrageenan (KC, molecular weight range 350,000–800,000 g/mol, Genuvisco type CI-102, lot # SKS2500) was donated by CP Kelco (USA). Multiwalled carbon nanotubes (MWNTs) produced by catalytic chemical vapour deposition were obtained from Nanocyl S.A. (Belgium, lot # 090901). Single-walled carbon nanotubes (SWNTs), produced by high-pressure decomposition of carbon monoxide (HiPCO process), were purchased from Unidym Inc. (USA, lot # P0348). Glycerin was obtained from Sigma Aldrich (USA, lot # 033K0097). Methanol (CH_3_OH, lot # 318-2.5L GL) was purchased from Ajax Finechem (Australia). Hydrophobic polytetrafluoroethylene (PTFE, pore size of 5 μm) filtration membranes were purchased from Micro Filtration Systems (USA). Milli-Q water was used in all experiments and prepared by using a Millipore filtration system (resistivity of 18.2 MΩ cm).

### Preparation of solution and dispersion

**Figure 9:**
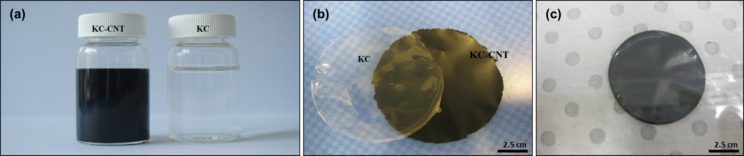
Photographs of (a) KC solution and KC–CNT dispersion, (b) films prepared by evaporative casting and (c) film prepared by vacuum-filtration method.

Solutions of KC were prepared by adding appropriate amounts of KC to 15 mL of Milli-Q water under stirring for 3 h at ~70 °C (Figure 9a). Homogenous KC–CNT dispersions (CNT concentration = 0.1% w/v, Figure 9a) were prepared by using a digital sonicator horn (Branson 450, Ultrasonics Corp.) with a probe diameter of 10 mm, in pulse mode (0.5 s on/off) and a power output of 12 W. During sonication the sample vial was placed inside a water bath to keep the dispersion temperature constant. Glycerin (G) was added to KC–CNT dispersions at a concentration of 0.25% w/v.

### Preparation of films by evaporative-casting method

Free-standing films were prepared by evaporative casting of KC solution and KC–CNT dispersions into the base of cylindrical plastic containers (polystyrene, diameter = 55 mm), which were then dried under controlled conditions (35 °C, relative humidity, RH = 45%) in a temperature–humidity chamber (Thermoline Scientific) for 24 h. The resulting films were peeled off the substrate to yield uniform free-standing films (Figure 9b).

### Preparation of films by vacuum-filtration method

KC–CNT dispersions were processed into buckypapers by using a vacuum-filtration method. Prior to the filtration the KC–CNT dispersion was combined with 35 mL Milli-Q water and inverted to ensure complete mixing. The dispersions (50 mL) were drawn through a PTFE membrane filter (pore size = 5 μm) on a filtration unit (Millipore, diameter = 37 mm) by using a vacuum pump (Vacuubrand CVC2). Once all of the dispersion had been filtered, the films were washed with 50 mL of Milli-Q water followed by 5 mL of methanol (99.8%) and placed between absorbent paper sheets to dry under controlled conditions (21 °C, RH = 45%) for 24 h. The films were then peeled off from the filtration membrane (Figure 9c).

### Characterization

UV–visible–NIR absorption spectra of KC solutions and KC–CNTs dispersions were obtained with a UV–vis–NIR spectrophotometer (Cary 500) by using a quartz cuvette (path length = 5 mm). All solutions and dispersions were diluted by a factor of 10. Dispersions were imaged by using an optical microscope (LEICA Z16 APO) fitted with a digital camera (LEICA DFC280) and Leica Application Suite (version 3.1.0 R1) software. Rheological testing was conducted by using a parallel-plate rheometer (Anton Paar–Physica MCR 301) with a 50 mm diameter probe head (cone angle 1°) at 21 °C. KC–CNT dispersions and KC solutions were analysed by using flow curves (viscosity and shear stress versus shear rate). The dynamic modulus was measured by using oscillatory strain sweeps at constant frequency.

For conductivity measurements, films were cut into strips 0.5 cm in width and 3 cm in length and contacted with copper electrodes (3M). Current (*I*)–voltage (*V*) characteristics were obtained by measuring the current using a digital multimeter (Agilent 34410A) under a cycling potential applied by a waveform generator (Agilent 33220A). *I–V* measurements were conducted under controlled ambient conditions (21 °C, RH = 45%) as a function of film length, by repeatedly cutting the end of the strip, contacting with the electrodes and remeasuring the *I–V* characteristics. Film thickness was determined with a Mitutoyo IP65 digital micrometer.

The mechanical properties of all films were obtained by using a dynamic mechanical analyser (DMA) Q800 (TA instruments). Measurements were carried out under ambient conditions (21 °C, RH = 45%) on rectangular strips (length = 10 mm) at a cross-head speed of 0.1 mm/min. Tensile strength, strain-at-break and Young’s modulus were determined from the maximum stress, the strain at failure, and the slope of the initial linear part of the stress–strain curve, respectively.

Scanning electron microscope (SEM) images were acquired by using a JEOL JSM-7500FA. Samples were prepared by mounting small pieces of films onto a brass stub (11 × 5 mm^2^) with double-sided, conductive carbon tape (Proscitech, Australia).

Contact-angle measurements were carried out by using the sessile drop method on a goniometer (Data Physics SCA20), which was fitted with a digital camera. The contact angles of 1 μL Milli-Q water droplets on the surface of the samples were calculated after 30 s by using the accompanying Data Physics software (version SCA20.1). The mean contact angle was calculated based on measurements performed on at least five water droplets.

The sensing properties of the films were investigated with a custom-built system [52]. The films are connected in series to a known resister (909 Ω) and a battery (4.91 V) to form a voltage–current–resistor (*V–I–R*) electrical circuit as a prototype sensor. The sensitivity of the sensors was characterised by using measurements of the voltage drop across the known resistor and film under different environmental conditions, i.e., as a function of temperature and humidity, and by exposure to different gases (H_2_ and CH_4_) at a concentration of 100 ppm in air. For all measurements, air was used as the carrier gas. The chamber volume (1000 mL) ensures that the change of gas concentration was instantaneous, which is a prerequisite condition for the accurate measurements of response and recovery time of the sensor.
